# “I would do something if I could!”: experiences and reflections from ethics teachers on how to respond when hearing alarming cases from medical students

**DOI:** 10.1186/s12909-021-02675-y

**Published:** 2021-04-23

**Authors:** Amalia Muhaimin, Maartje Hoogsteyns, Raditya Bagas Wicaksono, Adi Utarini, Derk Ludolf Willems

**Affiliations:** 1 Department of Bioethics and Humanities, Faculty of Medicine, Universitas Jenderal Soedirman, Kampus Kedokteran, Jl. Dr. Gumbreg 1, Purwokerto, 53112 Indonesia; 2 Department of Ethics, Law, and Humanities, Amsterdam University Medical Centre, University of Amsterdam, Meibergdreef 15, 1105 AZ Amsterdam, The Netherlands; 3 Amsterdam Public Health Research Institute, Amsterdam, The Netherlands; 4 Department of Health Policy and Management, Faculty of Medicine, Public Health and Nursing, Universitas Gadjah Mada, Yogyakarta, Indonesia

**Keywords:** Ethics teachers, Medical students, Clinical clerkship, Alarming cases, Student reports, Student disclosures

## Abstract

**Background:**

Previous studies show that teachers can feel disturbed by alarming cases brought up by students during their teaching activities. Teachers may feel uncertain about how to deal with these cases, as they might feel responsible to take action to prevent further harm. This study aims to explore how ethics teachers in medical schools would respond to a student report of unethical or unprofessional behaviour during the clinical training phase (clerkship) that is alarming and potentially harmful for patients or students themselves.

**Methods:**

This study used qualitative methods with purposive sampling. We conducted in-depth interviews with 17 teachers from 10 medical schools in Indonesia. We asked if they had heard any alarming and harmful cases from students and provided two cases as examples.

**Results:**

Four teachers shared their own cases, which they perceived as disturbing and alarming. The cases included power abuse, fraud and deception, violation of patient’s rights and autonomy, and sexual harassment. Regarding teachers’ responses in general, we found three main themes: (1) being assertive, (2) being careful, (3) barriers and facilitators. Most teachers were convinced of the need to take action despite numerous barriers, which they identified, leading to doubts and concerns in taking action. Our study shows that formal education in ethics might not necessarily influence how teachers respond to alarming cases, and that their responses are mainly influenced by how they perceive their role and responsibility as teachers.

**Conclusions:**

Our study suggests that teachers should carefully consider the risks and consequences before taking action upon alarming cases to prevent further harm, and that support from higher authorities might be crucial, especially in the Indonesian context. Our study also shows that taking action as a group might be appropriate in certain cases, while personal approaches might be more appropriate in other cases. Most importantly, school leaders and administrators should develop effective organisational culture and support students and teachers for their ethical responsibility commitment.

**Supplementary Information:**

The online version contains supplementary material available at 10.1186/s12909-021-02675-y.

## Background

Teachers in medical schools often hear reports of ethical problems and unethical practices in training sites. These reports may come from students or colleagues through formal or informal communications or mechanisms. In places where ethical case discussion is used as one of the teaching strategies, students sometimes present alarming cases that are potentially harmful for patients, healthcare workers, or students themselves. These may include breaches in medical ethics as well as unethical behaviour of healthcare workers towards patients and students. Previous studies show that both students and teachers can feel emotionally disturbed by ethical problems. Students often observe or encounter ethical issues or ethical dilemmas during their clinical training in the hospital and often experience moral distress [1–3]. Teachers, on the other hand, sometimes feel disturbed and uncertain about how to deal with cases that are brought up by students during their teaching classes [4]. Teachers may face a dilemma of weighing between the safety of patients in one hand and keeping the privacy and confidentiality of students on the other. As ethics teachers, they might also have limited authority to handle such cases. The development of systems to respond to student disclosures may vary among institutions. In some medical schools in Indonesia, for instance, there is a counselling unit that provides consultation for students who have academic problems. Teachers may also refer students to such unit if they feel the student is in need of psychological support. However, the counselling unit might have limited authority to deal further with cases involving potential ethical and professional misconducts in the hospital.

Medical ethics teaching is not something new in Indonesia. For decades, medical ethics (*etika kedokteran* - Indonesian) has been a mandatory subject in all medical schools. However, lectures in ethics were often limited to introducing the Indonesian Medical Code of Ethics or KODEKI *(Kode Etik Kedokteran Indonesia),* and occasionally, some existing law or regulations in health care. Lectures were usually given by senior professors, mainly medical specialists, without any formal background in ethics, although some might have had formal training in law or medico-legal. The lectures may have included examples of ‘ethical violations’, namely breaches or violations of the medical code of ethics or health law. However, in-depth discussions in class were rarely carried out. This condition was perhaps due to the limited time allocated within the medical curriculum and the previous learning methods in general, which did not have much room for discussions. Unlike *medical ethics* in this sense*, bioethics* is a new emerging field in the country; even though both share the same concept of addressing ethical issues in (bio)medicine and health care. For the Indonesian medical community, bioethics, which was widely introduced around the year 2000 in national conferences [5], has brought forward the idea of *ethical dilemmas* and *ethical principles,* thus opened space for *ethical discussions* within the medical curricula. In 2006, a new standard of competencies for physicians was introduced [6] along with a so-called competence-based curriculum (KBK) model and problem-based learning (PBL) method. Since then, medical schools have established competeny-based curricula and adopted the PBL method, which provided more room for in-depth ethical case discussions in large or small groups.

This paper demonstrates how ethics teachers in medical schools in Indonesia reflect on how to respond when they find out about alarming and potentially harmful cases from students during teaching. Our study aims to explore how teachers in medical ethics would respond to a student report of unethical or unprofessional behaviour during the clinical training phase (clerkship) that is potentially harmful for patients or students themselves. Knowing teachers’ responses, we will be able to identify what can and what cannot be expected from them and what kind of support is needed, especially regarding their positions as ethics teachers in an academic hospital. For this purpose, we conducted a qualitative study to explore what kind of alarming cases were brought up by students, teachers’ initial responses, and how they reasoned and reflected on their decisions. This study is part of a larger study on ethics education in medical schools during the clinical training phase (clerkship) in Indonesia and The Netherlands.

## Methods

This qualitative study used purposive sampling and thematic analysis. In 2018, there were 86 medical schools (36 public, 50 private), with one third located in Java [7]. However, information on the total number of ethics teachers from all medical schools was not available. Therefore, we first identified teachers who were actively involved in the development of bioethics and who have participated in bioethics meetings and training courses in Indonesia. We selected 25 potential participants, starting with teachers from leading medical schools that might have had ethics teaching in the clerkship phase. We then invited a diverse sample across teaching sites: both public and private universities, more recent medical schools, and diverse demographic characteristics, including age, gender, and educational background. We obtained teachers’ phone numbers and invited them through text messages, explaining the purpose of the study briefly and inviting them for an in-depth interview. Upon their agreement, we sent the information sheet and consent forms through e-mail. All teachers who were invited agreed to participate, but one participant eventually cancelled the interview due to other obligations. Due to the relatively small number of teachers working in this field, most participants were already familiar with the researcher professionally. The researcher’s professional backgrounds and experience in ethics teaching were most valuable in building rapport and gaining trust from participants. We believe that good rapport between the researcher and participants is essential for this study, considering sensitive matters that may come up during the interview.

The interviews were conducted at participants’ respective workplaces, except for one participant who preferred to be interviewed outside of her workplace. Three interviews were conducted by telephone due to the long distances. Permission to record the interview and take field notes was obtained. All data were kept anonymous and unidentifiable to ensure the teachers’ and students’, as well as the patients’ privacy and confidentiality. In-depth interviews were conducted by AM, RBW and DL in *Bahasa Indonesia* and transcribed verbatim. Transcripts were de-identified, meaning no personal identities and other potentially identifying information were written in the transcripts. Coding was done manually by AM and RBW, using excel sheets and tables. Initial codes were generated from teachers’ responses of alarming cases, how they reasoned and reflected on their decisions, and grouped into potential themes and sub-themes. Themes were checked against each other and back to the original data set. Potential themes and sub-themes, as well as naming of the main themes, were reviewed and discussed together with MH, DW, and AU (who did not conduct the interviews and did not know the respondents) until consensus was reached [8]. Data saturation was reached after 15 interviews, and two additional interviews were conducted to make sure no new themes emerged, adding up to 17 participants in total [9, 10]. The interviews’ duration ranged from 38 to 126 min, with an average of 80 min per interview. AM and RBW are medical doctors and teachers in medical ethics in Indonesia, while MH and DW are teachers in medical ethics in the Netherlands. AU is a medical doctor, professor of research methodology and qualitative methods in Indonesia, and is not involved in ethics teaching. The mixed team members from Indonesia and the Netherlands, with professional backgrounds and experience in both medical training and ethics teaching, were most valuable in the process of data analysis, in being able to relate well to the issues, in sharing insights and perspectives, and adding reflexivity to the process [10, 11].

We first asked participants if they had any experience in ethics teaching in the clerkship phase, and if they had, during their teaching activities, heard any cases from students which they thought were alarming and harmful. We then asked how they responded, if they had done any action outside the classroom, and asked their reasoning. We were interested in teachers’ personal responses and actions to any actual, reported or theoretical, student disclosures of alarming behaviours. Hence, we provided two cases from our previous studies as theoretical examples in case they were not involved in ethics teaching in the clerkship phase. The first case was about a student who was told to cover up mistakes in the operation room; the second was about a student who was asked to conduct physical examination of an intimate area on unconscious patients without consent beforehand for teaching purposes. We have chosen the two cases for two reasons. First, both cases presented potential harm and involved vulnerable patients, fraud, and deception. Second, both cases were considered disturbing in previous studies elsewhere [3, 4, 12]. We asked them what they thought if they were the teachers who received the cases, explored further if there were any actions they would have done, and asked their arguments. Interpretations of transcripts, including the English translations, were sent to participants through e-mail to ensure their own meanings and perspectives are correctly represented [13–15]. Two participants suggested minor corrections of translation, and one participant did not respond. No repeat interviews were carried out.

## Results

### Teachers’ characteristics

Seventeen teachers from ten medical schools in Sumatera, Java, and Sulawesi participated in our study. Fifteen participants were professionally trained as medical doctors, either with or without additional speciality training (referred here as ‘medical specialist’ and ‘general practitioner’). Most participants also had additional training (master and/or doctoral) in one or two of the following disciplines: medico-legal, ethics, philosophy, or medical education. Only two participants were not medical doctors and had formal educations in philosophy and ethics (Table 1).

**Table 1:** Teachers’ characteristics

Characteristics		Number
Sex	Female	8
	Male	9
Home base university	Public	12
	Private	5
Experience in ethics teaching	< 5 years	5
	5–10 years	5
	> 10 years	7
Professional background	Medical Doctor	15
	Medical specialist	9
	General practitioner	6
	Non-Medical Doctor	2
Additional master/doctoral degree	Medico-legal	6
	Ethics	3
	Philosophy	3
	Medical education	3

### Teachers’ stories

When asked if they had any experience in ethics teaching in the clerkship phase, only five (out of ten) medical schools in our study had some form of structured ethics teaching in the clerkship phase, and four (out of seventeen) teachers experienced receiving cases from students during their teaching activities. Hence, not all teachers were given the two examples of alarming cases (see Methods, third paragraph), as they had shared their own cases that had happened repeatedly and were considered potentially harmful and alarming. The alarming cases shared by those four teachers in our study included abuse of power, fraud and deception, violation of patient’s rights and autonomy, and sexual harassment. Below are two cases in which teachers took action, yet in a different way. The teachers shared their opinions about the outcome, what went well and what could have been done differently.

#### Taking advantage from students

One of the teachers shared a case from her colleague about a student who felt uncomfortable working at one of the clinical departments because one specialist sometimes asks students to do things that were not part of their tasks.

*“The student said that the doctor sometimes asked students to take him somewhere, buy some food, or pick up his kids. They were also told to work at a hospital they had no MoU with. When we tried to investigate further, another student mentioned: ‘Well, 200 or 300 thousand rupiahs might mean a little to others, but it means a lot for us because we have to pay’.”* ***(G016)***

The case became a heated topic because the teacher immediately reported the case through a *social media group for teachers* mentioning the student’s name. The case was reported further by the head of the medical program to the head of the clinical department and the dean. The doctor was then identified and questioned by the authorities: *“What have you done, telling students to do things that are not part of their job?”* The doctor who was accused said that he felt mistreated and humiliated. Some students said that the doctor should not have been reported because it was common in medical training. After the incident, students became hesitant to share cases and became quiet during discussions due to fear of being reported.

When asked what she thought about the action taken, the teacher said that it would have been better if they had met the doctor in person to confirm the case and remind him in a nice way. They should also not mention any names, including students, to protect one’s reputation and not ruin the relationship between teachers and specialists at the hospital. Although some of her colleagues thought differently, saying that it was appropriate to open the case to prevent others from secretly doing such a thing, she disagreed and supported other colleagues who were worried that the specialists would keep a distance and would no longer be willing to supervise students; and that would not be good for the students and the institution.

#### Asking for extra payment from patients

Another teacher shared a case which he heard from his students during the ethics discussion. The students said that one of the doctors in the hospital charges extra payment from patients. He was shocked and thought that it was a crime to do such a thing.

*“There is this doctor who charges extra payment from patients, where in fact the cost should be paid to the hospital administration. When the student asked the doctor (the doctor replied): ‘This is my USG (Ultrasonograph) device, it does not belong to the hospital’. Students did not consider it as unethical but unprofessional, and some even said it’s a crime. I think it is a crime.” (G007)*

He decided to collect more data and discovered that similar cases had happened. Together with colleagues who were in charge for the clerkship program, he reported the case to the higher authorities at the faculty level, who then conducted further investigation in the hospital. They believed that the case needed to be dealt with carefully, so an in-house training for all doctors was organised to protect the anonymity of the doctors involved. The case was re-written as if it happened elsewhere with a different nuance. The organisers also ensured that participants could identify or relate to the case and let them know what the authorities and ethics team thought about it. Participants then responded by mentioning that they had done similar things in their practice.

The teacher said that he was happy with his action because when they discussed the issue together, the doctors became aware that it was unethical to do such a thing, mostly because they sometimes had to falsify certain documents. This issue includes cases of double insurance, where they had to falsify documents based on the patient’s request. The teacher thought it was good if doctors could have such discussions and have some ‘mutual awareness’ about ethical problems. Through teachers’ narratives, we identified actual actions taken in various steps and forms (Table 2). These actions were taken outside of class, namely outside of students’ learning environment and learning process.

**Table 2:** Actual actions taken

Participants	Alarming cases	Actions
G001	Disrespectful to patients and violation of patients’ rights	Investigate further Personal approach
G007	Fraud and falsification of financial reports	Investigate further Pass information to the higher authority Collaborate with other units/departments Educate doctors through workshops/seminars
	Sexual harassment to medical students	Investigate further Pass information to the higher authority Personal approach
G015	Deception and violation of patients’ rights	Investigate further Collaborate with other units/departments
	Misconception leading to breaches in medical ethics	Conduct extensive study/research Educate doctors through workshops/seminars
G016	Abuse of power to medical students	Pass information to the higher authority Discuss openly in departmental forum/meeting

### Teachers’ responses

**Fig. 1:**
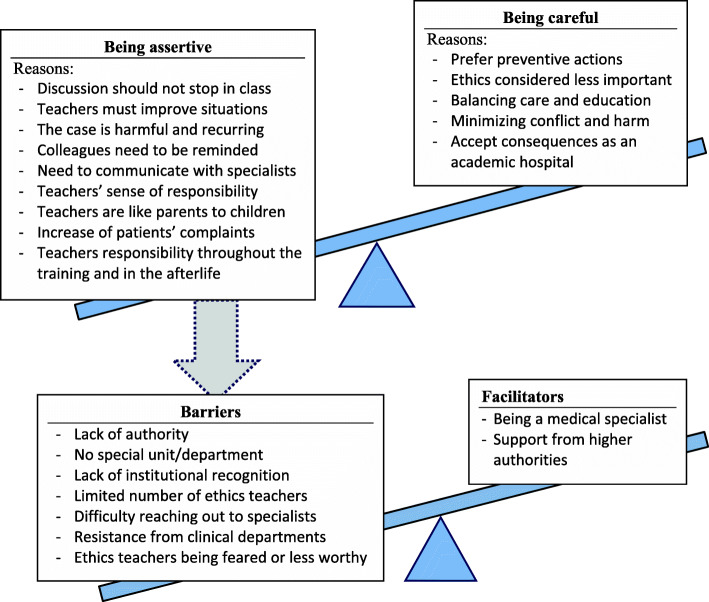
Teachers’ responses

We explored responses from all seventeen participants in our study, even though only four teachers had their own cases from students’ reports. For the other teachers who did not have any cases from students’ reports, we provided the two alarming cases from our previous study. Although most of the teachers (14 out of 17) in our study were convinced of the need to take action when hearing alarming cases, they all shared concerns about doing so after reflecting and identifying the barriers which were more prevalent within the training system compared to the facilitators. We came up with three main themes: [1] being assertive, [2] being careful, [3] barriers and facilitators (Fig. 1).

#### Being assertive

Most of the teachers in our study were quite assertive in saying that further action should be taken when it comes to patients’ and students’ safety and well-being. Below are responses from two lecturers who had reasons to believe that action should be taken. The first lecturer, who was a general practitioner and relatively junior in terms of age and teaching experience, received a case about a doctor who blamed a patient in front of other patients for refusing treatment, pointing out that she was covered by the national health insurance, namely BPJS/JKN *(Jaminan Kesehatan Nasional),* meaning that *she was poor* and that she should just follow the doctor’s suggestion. The teacher felt disturbed by the fact that the doctor associated the JKN with poor people and that they were supposed to have less autonomy. She felt concerned that this false perception would spread out to patients and students.

*“It was really unethical and harmful for patients, so I asked the students to give a clue who the doctor was. It was not only because I was annoyed (geregetan -* Indonesian*), but I felt that I need to remind that person. I was hoping… if I knew the doctor personally, perhaps I could somehow communicate the problem, maybe indirectly... Perhaps we can discuss it.” (G001)*

The teacher wanted to approach the doctor but eventually decided not to, after discovering that she did not know the doctor well enough to discuss the sensitive matter. The second teacher, a senior medical specialist and head of a department, did not have any case of his own, but he had strong opinions in response to the case examples (see Methods section). The teacher did not hesitate in saying that doctors should be given sanctions to avoid further harm to patients, especially if they had been given some warning before, and there are no improvements. Nevertheless, he suggested that the cases should be first discussed within the clinical departments to avoid open conflicts.

*“We should talk to the head of the department. That would be the best way, although I have never done it before. If there were such cases, I would do it. If there were, for instance, a resident involved, we have to prevent harm to anyone, including residents. But if we cannot ‘fix’ them, then what to do, it’s harmful to patients! They might even need to be expelled from their work.” (G011)*

Among 14 teachers who were convinced of the need to act, almost half (6 teachers) were senior lecturers with more than 15 years of teaching experience (not only ethics in particular), and more than half (8 teachers) were non-specialists. Our study did not find any differences between junior and senior lecturers or between medical specialists and non-medical specialists in their willingness to act upon students’ reports of alarming cases.

#### Being careful

Only three teachers, all medical specialists, were less assertive and more careful in deciding to take action. Below are their responses on the case examples, as they had not received alarming cases from their students. The two case examples were about students who were told to cover up mistakes and asked to conduct physical examination of intimate areas on unconscious patients. They viewed the cases as rather dilemmatic situations in clinical training, and emphasised the need to carefully balance the values, risks, and consequences to avoid further harm to students, patients, and doctors.

*“It is dilemmatic. I think we need to analyse it further because I don’t know... How is it actually from an ethical perspective? If it is not considered right, then clinicians should be informed. Maybe they are not fully aware and just want to educate students.” (G013)*

*“I cannot blame nor justify anyone. How can they (students) have clinical skills if they do not examine patients? We must introduce them, and many patients might refuse, so maybe that is the dilemma. If all patients refuse, then what will happen to our students?” (G010)*

*“It is a win-win solution because the learning process needs to go on... For those (students) who feel it is conflicting with their conscience, then they should not do it, but they should not get punished (for not following orders). But if they are willing to follow, then they may.” (G014)*

One explanation that may be generated from the interviews and the quotes above was that medical specialists had experienced the complicated situation of being a clinical teacher in the hospital with dual responsibility towards patients and towards students. This complexity might explain why they were more careful in balancing between what is best for their patients and what is best for students’ learning experience.

#### Barriers and facilitators

In our study, teachers identified different barriers and facilitators, despite their strong willingness and intentions to act upon hearing the alarming cases. Two main facilitators for taking (or suggesting to take) action were: [1] being a medical specialist (clinician) and [2] support from higher authorities (see Fig. 1: Facilitators, and Table 3). The latter was considered most effective in implementing actions, although being a specialist was considered more influential in promoting ethics and spreading the knowledge among other specialists in the hospital.

**Table 3:** Facilitators to take action

Coding	Quotations
Being a medical specialist	*“So, the clinicians… when they see you (as a general practitioner), they would say: ‘you’re not a clinician, so why do you say such things?’ But if I (as a clinician) say it, then they will be surprised!” (G010)*
Support from higher authorities	*“I think the best way for medical schools in Indonesia is a top-down approach. I think what I did previously with the bottom-up approach was quite aggressive, but if there is no structure (authority), then it’s not convincing...” (G017)*

Nevertheless, the majority of teachers shared similar concerns and barriers for the overall situation of ethics teaching in medical schools, in the clerkship phase in particular (see Fig. 1: Barriers), especially the difficulty to reach out to their colleagues in the clinical departments (referred to as “specialists” or “clinicians”). Other barriers include having less authority, the limited number of ethics teachers, and lack of institutional recognition (Table 4).

**Table 4:** Barriers to taking action

Coding	Quotations
Difficulty reaching out to specialists	*“I think it’s difficult… rather impossible in the medical culture. It’s an institutional problem. It is strange, indeed, this relationship (between non-specialists and specialists). The specialists sometimes don’t think of themselves as teachers.” (G003)*
Resistance from clinical departments	*“Maybe because they (clinicians) are old, so they have a different way of thinking. And the problem is that many of them do not like ethics; so (they would say) ‘why should we bother with such thing?’ I think.” (G010)*
Being feared or less worthy	*“Well, I would (do something) if I could! But the problem is… I’m not sure, maybe this is just a coincidence, but I think I’m not a likeable figure here… maybe they are afraid of me, or just reluctant, I don’t know…” (G014)*
Difficulty reaching out to specialists Resistance from clinical departments	*“We need to remind the students, that is most important! Ask them what they think about it. But If we want to intervene in the (clinical) departments, it would be very difficult, because as you know, they are like these ‘kings in small kingdoms’, right?” (G015)*
Lack of authority Limited number of staffs Lack of institutional recognition	*“I think I cannot do it alone. I was no longer head of the bioethics team, so I have to say that the case was rather neglected because I need a partner to work with, someone who is also interested in ethics. At this moment, we only have six people in this department (which is not an ethics department), each with a different specialisation… If there are not any separate body/unit and at least 1–2 people focused on ethics, then it becomes difficult” (G017)*

These barriers were mentioned by most teachers in our study. Moreover, we did not find any major differences among groups of teachers, i.e. between teachers with different educational backgrounds, between medical specialists and non-medical specialists, or between junior and senior lecturers. In Table 4, three of the respondents were senior medical specialists with high positions within their respective institutions, who presumably would have had more authority compared to other teachers. Nevertheless, they shared and identified similar concerns and barriers with regard to ethics teaching in the clinical clerkship phase.

## Discussion

### Being assertive: responsibility as teachers

The majority of teachers in our study were assertive in responding to both actual and hypothetical student disclosures of alarming cases. In Indonesia, teachers view their tasks as to transfer knowledge and skills, and guide students throughout their education. Teachers are not only responsible for students, but also responsible to the parents, nation, and religion [16, 17]. Although teachers in our study are classified as ‘lecturers’ (*dosen -* Indonesian), they perceive their role and responsibility as ‘teachers’ (*guru -* Indonesian). The law states that both teachers and lecturers should commit to promoting faith, piety, and noble character [18]. This role is supported by the Standard of Competencies for Indonesian Physicians (SKDI), placing “Noble Professionalism” as the first and basic area of competence, which includes belief in God, ethics, and discipline [19]. Teachers’ responses in our study reflect this view, saying that they are responsible for students, as parents to children, throughout their training and until the afterlife. This view might explain why teachers’ initial responses were quite assertive in taking action, considering the barriers they were aware of. However, our findings might also suggest how ethics teachers in general respond to reports of alarming cases from colleagues or students outside of their teaching activities. Moreover, clinical educators who are not involved in ethics teaching might also have similar responses when hearing alarming cases from students.

Indonesia is experiencing a transition in medical education and health care. Ethical issues in medical training and health care practices are often related to violations of the country’s medical code of ethics (KODEKI). Indonesia’s journal of medical ethics or *Jurnal Etika Kedokteran Indonesia* (JEKI), published by the Medical Ethics Honorary Board (MKEK) and launched in 2017, is nuanced with topics and discussions of malpractice and ethical misconducts [20–22]. According to MKEK, Indonesia has experienced the so-called “malpractice fever”, where there were 122 cases reported within two consecutive years (2004–2005), with at least one-third involving suspects of malpractice, medical error, and legal disputes between doctors, as well as between doctors and hospitals and other professions. Since then, their work and focus has been on professionalism, including ethics and law, to regulate and enhance professionalism of Indonesian doctors with a so-called “ethico-legal” system [23]. Moreover, Indonesia has recently implemented its national health insurance (JKN), dealing with problems of inequity and social justice, and an increasing number of complaints from both patients and healthcare workers [24]. In our study, teachers shared deep concerns, implying a burden and struggle in teaching ethics to future medical doctors in a rather complex and intrusive system. However, they showed enthusiasm in fighting for the rights of patients, as well as students and physicians. Perhaps we can understand the willingness of most teachers in our study to take action, despite the noted barriers, in relation to this struggle and the emergence of bioethics as a new field in Indonesia.

### Being careful: identifying barriers

The medical profession in Indonesia is considered noble and exclusive and enjoys high social status. Therefore, teachers in a medical school without any medical background may feel intimidated if they are involved in clinical discussions. There is also a gap between general practitioners and medical specialists, although both have medical backgrounds. General practitioners are somehow perceived as having a lower degree and social status within the medical field. Moreover, doctors are trained in a hierarchical and authoritative system, often causing negative emotions and barriers to communicate [25–27]. Among seventeen teachers in our study, nine were medical specialists. In contrast to what the above literature suggests, our specialists also identified numerous barriers and shared reasons and doubts not to take action. This phenomenon is perhaps due to the fact that ethical cases came from the clinical departments, and specialists were aware of the reluctance and resistance from their own colleagues. Another reason could be that medical specialists working in academic hospitals have a dual role and responsibility, as a physician who provides care in the best interest of the patient, and as a teacher who carries the responsibility to educate students and share their knowledge and clinical skills with medical students. Our findings show how they reflected on the complexity of being clinical teachers, balancing between their responsibility to patients and students, as well as dealing with their colleague specialists, resulting in a more careful response. Hence, our study suggests that being a specialist is sometimes not enough to facilitate action, although they are considered to have a large influence in introducing ethics to other specialists.

In Indonesia, not all medical schools have teachers trained in ethics, although ethics is in the curriculum. Medical ethics has often been referred to as the medical code of ethics. Hence, ethical problems are often perceived or related to ethical misconducts and violations of the code of ethics. Therefore, ethics teachers are sometimes feared by other teachers for monitoring or criticising their behaviour. This fear is understandable when ethical problems are related to issues of malpractice and ethical misconduct. Ethics is also considered less important and therefore has limited time and space within the curriculum. These reasons might explain why ethics teachers sometimes do not feel appreciated by their colleagues for the ethical knowledge they have. Hence, pursuing a career in ethics becomes less appealing, potentially causing the limited number of teachers specialised in ethics. This condition could bring further concerns and consequences, including not being acknowledged, not having an official body/unit, and lack of institutional recognition. We believe that this problem should be resolved to prevent teachers from being discouraged in learning and teaching ethics. In our study, three teachers had formal education in ethics, and the majority have followed some ethics training. However, our study does not show any differences in responses between teachers who have and do not have formal education in ethics. This finding indicates that formal education might not necessarily influence how teachers respond to alarming cases, and that their responses are mainly influenced by how they perceive their role and responsibility as teachers and by the culture and environment in which they work.

### Teachers’ action: balancing risks and consequences

One of the cases told in our study (see Results: Teachers’ stories) describes an individual action taken spontaneously by a teacher who reported the case to the higher authorities. It was somewhat unclear if the incident had any positive outcomes or consequences and caused any changes in the behaviour of the doctor(s). Nevertheless, there were negative consequences for the accused person and other students who had taken the doctor’s blame. Fortunately, there were no consequences for the student who reported the case. Previous studies suggest that such individual actions, often associated with whistleblowing, may cause negative consequences [28–30], especially in cultures where group loyalty and harmony are important values [31]. Furthermore, spontaneous actions taken without careful considerations might cause harm, especially for students who are in a vulnerable position within the medical training system. Ciasullo (2017), therefore, suggests that whistleblowing *“should be understood as a collective, social, and cultural action rather than an individual initiative”* [32]. However, individual actions using personal approaches might be appropriate in certain cases, where professional relationships and closeness become an advantage to discuss sensitive ethical problems openly.

In the second case, careful steps were taken before bringing the case to the higher authorities, and the final action was done together as a group with the authorities. Most importantly, the action was targeted to a group of people and therefore minimising harm to individuals. This case shows that discussing cases and concerns with other teachers might be crucial before deciding to take further action. Furthermore, taking action as a group with shared responsibility might be safer and more ‘convincing’ for higher authorities to accept, as well as for the targeted people [33]. Although higher authorities were involved in both cases, we learned that it is crucial to carefully consider the final goal and most appropriate way to achieve it while preventing further harm. The downside from this non-direct and *multi-level* approach is that the process might take more time and bring uncertain results, while the alarming ethical problem remains, thus potentially causing harm to other individuals. Balancing risks and consequences, therefore, becomes crucial in such cases. Our suggestions, however, are based on these two cases. More research on situations in which action was taken is needed to get a clearer picture of what kind of support is needed by teachers in this matter.

### Recommendations

Finally, we suggest that in the context of ethics education, it may be useful to set up technical procedures for safe reporting mechanisms for both students and teachers. Medical schools can provide consultation for teachers through independent advisors, or advisory boards, to deal with alarming cases for the sake of patients and students, while maintaining privacy, confidentiality, and protecting all parties from blame and further harm [34]. In the hospital setting, ethics committees perhaps could play a role in facilitating openness about alarming cases. However, these recommendations might differ between institutions and regions in Indonesia and in other countries, taking into account different sociocultural factors and educational systems [35, 36].

### Strengths and limitations

The selection of participants was based on our network with individuals and institutions who have collaborated and participated in ethics educational programs in Indonesia, mainly coming from Java, Sumatra, and Sulawesi. There might have been teachers and medical schools elsewhere in Indonesia who have already conducted ethics teaching in the clerkship phase but were not included in our study. Although back-to-back translations from Bahasa Indonesia to English were carefully done for the quotations and interpretations, some words might have slightly different meanings and be perceived differently by non-Indonesian readers. To our knowledge, there have not been any similar studies regarding this topic in the Indonesian context. Numerous studies have been conducted elsewhere on ethics education and students’ experiences in dealing with ethical issues and moral dilemmas. However, they rarely (if not any) discussed teachers’ experiences and dilemmas in dealing with students’ disclosures of alarming cases. We hope this study can contribute to the development of medical ethics education in Indonesia and in other countries.

## Conclusions

Our study provides an insight into how ethics teachers in medical schools in Indonesia reflect and respond to ethical cases that were alarming and potentially harmful. Teachers were assertive and expressed a strong willingness to act. However, teachers also identified numerous barriers from within the educational system and medical profession, causing doubts and concerns to realise their actions. We suggest that medical schools and academic hospitals should facilitate clinical teachers and teachers in ethics to discuss their concerns. Our study also shows that in such a high-context and collective culture, taking action as a group with shared responsibility and targeting groups instead of individuals might be appropriate in certain cases to prevent further harm. However, personal approaches might be necessary in cases where close professional relationships can facilitate an open dialogue and discussion on sensitive matters. Most importantly, school leaders and administrators should develop effective organisational culture and support students and teachers for their ethical responsibility commitment.

## Supplementary Information

**Additional file 1.** Interview guide

## Data Availability

The datasets generated and/or analysed during this study are not publicly available due to privacy and confidentiality reasons but are available from the corresponding author on reasonable request.

## Abbreviations

BPJS: Badan Penyelenggara Jaminan Sosial
JEKI: Jurnal Etika Kedokteran Indonesia
JKN: Jaminan Kesehatan Nasional
KODEKI: Kode Etik Kedokteran Indonesia
MKEK: Majelis Kehormatan Etik Kedokteran
SKDI: Standar Kompetensi Dokter Indonesia
