# Supplementary material for: “I would do something if I could!”: experiences and reflections from ethics teachers on how to respond when hearing alarming cases from medical students
Source: BMC Med Educ. 2021 Apr 23;21:233. doi: 10.1186/s12909-021-02675-y (PMC8067644; doi:10.1186/s12909-021-02675-y)
Supplement: Supplementary file 1 — Additional file 1. Interview guide [file 12909_2021_2675_MOESM1_ESM.doc]

**Interview guide**

**Code:**

|  |
| --- |

1. Could you tell me a little about your background?

- Formal education
- Teaching experience

1. Are you involved in ethics teaching in the clerkship phase?
2. (If **YES**) Could you describe how the teaching is organized in the clerkship phase?

- Structure / organization
- Learning methods / strategies

1. Students sometimes share alarming cases (harmful for patients, students, or healthcare workers) from their training during ethics discussions.
   1. Do you have such experience?
   2. How did you feel about the case?
   3. What actions have you done (or would have done)?
   4. What are the reasons for acting/not acting upon the case?
2. If you are not involved in ethics teaching in the clerkship phase or do not have such experience, here are two case examples from our previous study:
   - A student who was told to cover up mistakes that occurred in the operation room and keep quiet (not disclose to anyone)
   - A student who was asked to conduct physical examinations to unconscious patients, without obtaining consent beforehand for teaching purposes

Questions:

1. What do you think about the cases?
2. Would you or would you not act upon the case?
3. What are your reasons for doing or not doing so?
